# Supramolecular Arrangement of Lignosulfonate-Based Iron Heteromolecular Complexes and Consequences of Their Interaction with Ca^2+^ at Alkaline pH and Fe Plant Root Uptake Mechanisms

**DOI:** 10.1021/acs.jafc.3c03474

**Published:** 2023-07-18

**Authors:** Marta Fuentes, German Bosch, David de Hita, Maite Olaetxea, Javier Erro, Angel Ma Zamarreño, Jose Ma Garcia-Mina

**Affiliations:** † Universidad de Navarra, Instituto de Biodiversidad y Medioambiente BIOMA, Irunlarrea 1, 31008 Pamplona, España; ‡ Universidad de Navarra, Facultad de Ciencias, Departamento de Biología Ambiental, Irunlarrea 1, 31008 Pamplona, España

**Keywords:** iron chelates, heteromolecular iron chelates, heteromolecular iron complexes, iron chlorosis, root iron deficiency responses, transcriptional control, post-transcriptional control, Fe(III) chelate reductase, H^+^-ATPase

## Abstract

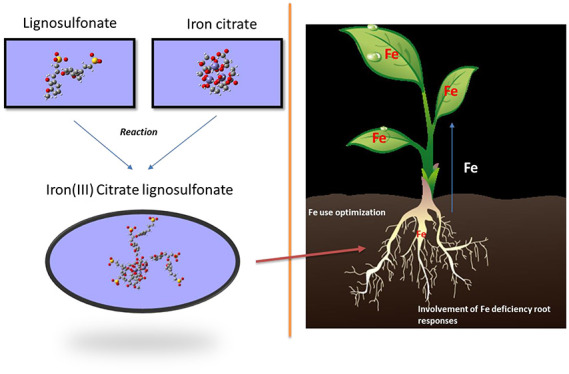

Previous studies have shown that natural heteromolecular complexes might be an alternative to synthetic chelates to correct iron (Fe) deficiency. To investigate the mechanism of action of these complexes, we have studied their interaction with Ca^2+^ at alkaline pH, Fe-binding stability, Fe-root uptake in cucumber, and chemical structure using molecular modeling. The results show that a heteromolecular Fe complex including citric acid and lignosulfonate as binding ligands (Ls-Cit) forms a supramolecular system in solution with iron citrate interacting with the hydrophobic inner core of the lignosulfonate system. These structural features are associated with high stability against Ca^2+^ at basic pH. Likewise, unlike Fe-EDDHA, root Fe uptake from Ls-Cit implies the activation of the main root responses under Fe deficiency at the transcriptional level but not at the post-transcriptional level. These results are consistent with the involvement of some plant responses to Fe deficiency in the plant assimilation of complexed Fe in Ls-Cit under field conditions.

## Introduction

Despite the high abundance of iron (Fe) in soils, its low solubility at alkaline pH and calcareous soils makes it highly unavailable for plants and microorganisms living in these soil types.^1^ This fact is related to the development of Fe deficiency in many crops, which is expressed as an interveinal yellowness commonly known as iron chlorosis.^1^ If this deficiency is not corrected adequately, a substantial impairment in both yield and quality takes place.^1^

Although different methodologies have been developed to prevent and correct iron chlorosis, the results showed that the most efficient strategy is using synthetic iron chelates with high stability and solubility in the soil solution of alkaline calcareous soils.^1,2^ Among them, the chelates that showed higher efficiency under very demanding conditions (high reactive soils) and perennial crops are the ortho–ortho Fe-EDDHA isomer or, more recently, Fe-HBED.^2−4^ Fe-EDTA is also employed under less demanding conditions or in the fertigation of vegetables.^2^ Despite the high efficiency of these chelates, their use poses some relevant problems. On the one hand, these chelates are very persistent in soils and can appear in leachates.^5^ On the other hand, some studies revealed that plant roots can take up these compounds and further translocate to the shoot.^6^ This fact might also raise nutritional and environmental concerns.

It has been proposed that several alternatives to synthetic chelates correct iron chlorosis. These alternatives include biodegradable synthetic chelates,^7^ siderophores,^8−10^ complexes with protein hydrolysates,^11^ humic substances (HS),^12−15^ and lignosulfonates (Ls).^16,17^ These more environmentally friendly solutions showed enough efficiency in low- and medium-demanding plant–soil systems as potential alternatives to Fe-EDTA.^7−17^ However, under highly demanding conditions (Fe-sensitive perennial crops, high active soil calcium carbonate, and alkaline pH), these Fe compounds have less efficiency than Fe-EDDHA in providing plant-available Fe to the soil solution.^18^ It is therefore necessary to increase the stability of these types of natural iron compounds. Our group has worked on preparing heteromolecular Fe chelates or complexes^19−21^ in this framework. Our hypothesis is that the consecutive use of two different ligands may favor the formation of more Fe-ligand bonds and prevent the formation of molecular aggregates through metal bridges, thus increasing their stability and solubility.^19−21^ In this framework, we obtained heteromolecular complexes based on Fe-citrate and HS (Cit-Fe-HS)^19^ and Fe-citrate and Ls (Ls-Cit),^20^ which showed a capacity to maintain Fe in solution at basic pH and in the presence of Ca^2+^ at higher concentrations than the corresponding Fe-citrate, Fe-HS or Ls, and Fe-EDTA as well.^21^ Other studies confirmed these results.^22^

Studies under field and highly demanding conditions (citrus trees cultivated in alkaline calcareous soil in the Murcia region) showed that a heteromolecular iron complex involving Fe-citrate and Ls (Ls-Cit) was able to correct Fe deficiency at the same level as that of Fe-EDDHA but probably through different mechanisms.^21^ Citrus trees treated with Ls-Cit showed lower chlorophyll in leaves than those treated with Fe-EDDHA at the beginning of the plant cycle.^21^ Likewise, the concentration of active Fe in leaves was higher in Fe-EDDHA-treated plants.^21^ However, chlorophyll leaf concentrations at the end of the cycle were similar in the Ls-Cit and Fe-EDDHA treatments and higher than the control (non-Fe treated) trees.^21^ A similar result was obtained regarding the final yield.^21^ As the concentration of active Fe in leaves was lower in Ls-Cit treated plants than in Fe-EDDHA treated plants, but yields and quality were similar, the fertilizer efficiency of Ls-Cit was higher than that of Fe-EDDHA.^21^ These results were also obtained in peach and orange orchards cultivated in alkaline and calcareous soils.^23^ In all cases, the efficiency of Ls-Cit increased with the use of a small amount of Fe-EDDHA (20% of total Fe applied) as a starter.^21,23^ These results suggest that this type of heteromolecular complex could be an ecological alternative to synthetic chelates. However, the mechanism of action of these heteromolecular complexes remains poorly understood and needs further study.

In this context, the above-described results are consistent with the hypothesis that the action of the heteromolecular complex involves the participation of the specific mechanisms that dicotyledonous plants have to optimize Fe use efficiency and cope with Fe deficiency.^23−27^

In order to investigate this hypothesis, we have carried out the following studies combining experiments dealing with Fe stability, the structural features of the Fe binding site in the Ls-Cit complex, and complexed-Fe root uptake mechanisms:

(i) The interaction with Ca^2+^ as a function of pH of Ls-Cit, Fe-citrate (Cit), and Fe-lignosulfonate (Ls-Nit).

(ii) The Fe deficiency root responses in cucumber plants treated with Ls-Cit and Ls-Nit complexes, and Fe-EDDHA. These root responses in dicots involve several complementary actions: the activation of a root-chelate reductase, the synthesis and activity of a Fe(II) transporter, the release of reductants and complexing agents such as coumarins and riboflavins depending on the plant species, and the activation of root H^+^-ATPase to acidify the rhizosphere.^23−25^ These responses are codified by specific genes that are regulated by several transcription factors.^26,27^ In our study, we have investigated root Fe(III)-chelate reductase (FCR) activity; rhizosphere acidification; the genes *CsFRO1* codifying FCR, *CsIRT1* codifying Fe (II) transporter, *CsFIT* codifying the transcription factor FIT, and *CsHA2* codifying root H^+^-ATPase; and genes codifying riboflavin synthesis: *CsRIBA1*, *CsRIBC*, *CsPYRD*, *CsPHS1*, and *CsDMRLs*.^28^ Riboflavin release to the rhizosphere was evaluated by fluorescence.^29^ This study has been complemented by determining the root concentration of IAA as a marker of the activation of Fe deficiency root responses.^30−32^

(iii) The stability and complexation degree of Fe in the Ls-Cit and the Ls-Nit complexes using a fluorescence quenching approach.

(iv) The structural features of Fe complexation in Ls-Cit and the Ls-Nit complexes using molecular modeling.

## Materials and Methods

### Preparation of Iron Complexes

Complexes with different Ls-C (carbon from Ls):Fe stoichiometries were prepared as follows: Ls was dissolved in water, and specific amounts of Cit or Fe(NO_3_)_3_ solutions were slowly added to the Ls solution, maintaining the pH at 8 with KOH. Solutions were left stirring overnight and eventually centrifuged at 3500*g* for 15 min. Final concentrations of Fe in supernatants were close to 5 g kg^–1^.

### Study of Iron Binding Stability in the Presence of Ca^2+^ and As a Function of pH

A 500 mL solution of 20 mg L^–1^ Fe was prepared for each complex. These solutions were divided into four portions of 120 mL, and the pH was adjusted to 7, 8, 9, and 10, respectively, with NaOH or HCl. Subsequently, those solutions at different pH’s were divided into two aliquots of 50 mL, adding 0.5 mL of 2 M CaCl_2_ to one of them (pH+Ca) and nothing to the other. Solutions were kept in the dark, and after 3, 7, and 14 days, 10 mL aliquots were filtered through a 0.45 μm pore-size syringe filter and the iron content in the filtrate was analyzed by ICP-OES.

### Study of the Main Root Responses to Fe Deficiency in Cucumber Plants Fed with Fe-EDDHA and Fe Complexes

#### Plant Materials and Growth Conditions

Cucumber seeds (*Cucumis sativus* variety Ashley) were germinated on filter paper in trays with perlite, watered with 1 mM CaSO_4_ for 7 days in a germination chamber at 25 °C, 75% relative humidity, and darkness. Subsequently, the seedlings were transferred to a hydroponic system in a growth chamber. The hydroponic system consisted of containers filled with 7 L of constantly aerated nutrient solution (NS). The composition of the NS is formed by the following elements: 2 mM Ca(NO_3_)_2_·4H_2_O; 0.75 mM K_2_SO_4_; 0.65 mM MgSO_4_·7H_2_O; 0.50 mM KH_2_PO_4_; 50 μM KCl; 1 μM MnSO_4_·H_2_O; 0.50 μM CuSO_4_·5H_2_O; 0.50 μM ZnSO_4_·7H_2_O; 0.35 μM Na_2_MoO_4_·2H_2_O; 10 μM H_3_BO_3_; and 1 μM Fe-EDDHA ([Fe(III)-EDDHA, 85% ortho–ortho isomer). The NS was renewed every 3 days and adjusted to pH 6 with 0.1 M NaOH. The growth conditions were 25/21 °C and 70/75% relative humidity in light/dark periods, with a photoperiod of 15 h of light per 9 h of darkness and a light intensity of 250 μmol m^–2^ s^–1^. After 7 days, NS was renewed (same composition as above but for iron), adding Fe as follows: (i) Fe^–^: negative control, 0 μM Fe; (ii) Fe^+^: positive control, 40 μM Fe-EDDHA; (iii) Ls-Cit: 40 μM Fe as lignosulfonate-iron-citrate complex; (iv) Ls-Nit: 40 μM Fe as lignosulfonate-iron; (v) Ls: 40 μM Fe as Fe-EDDHA and the equivalent amount of Ls added to Ls-Cit and Ls-Nit treatments; (vi) Cit: 40 μM Fe as Fe(III)-citrate. Each treatment has three containers (three repetitions) with 14 plants each, which are harvested at 1, 3, and 6 days after applying the respective treatments. The leaf chlorophyll index was monitored daily with a DUALEX Force-A optical leaf clip meter on the first true leaf of the plants with two measurements per leaf.

#### Ferric Chelate Reductase Activity (FCR)

Root FCR activity was measured as described in Bacaicoa et al.:^31^ 1 g of apical roots (five replicates per treatment) was immersed in 5.25 mL of NS (pH 6) containing 0.387 mM Fe(III)-EDTA and 0.286 mM bathophenantrolinedisulfonate (BPDS). BPDS binds to Fe (II), forming a reddish complex, whose concentration was determined after 30 min of incubation in the darkness and at room temperature by measuring the absorbance of the solution at 525 nm using an Agilent 8453 spectrophotometer with UV–visible Chemstation Software (Agilent Technologies, Santa Clara, CA, United States) using an extinction coefficient of 22.1 × 10^–3^ mM^–1^ cm^–1^.

#### Nutrient Solution Fluorescence

Exudation of riboflavin and other fluorescent compounds was estimated by fluorescence spectroscopy.^29^ Aliquots from NS were sampled daily, and fluorescence emission spectra were recorded on an RF-6000 fluorometer (Shimadzu, Kyoto, Japan) using an excitation wavelength of 370 nm.

#### Mineral Analysis

A digestion of the previously dried and ground plant material is carried out to determine the elements in the aerial part. Around 0.2 g of the sample is accurately weighed to be digested with 6 mL of 65% HNO_3_ and 2 mL of 33% H_2_O_2_, using a microwave oven ETHOS UP (Milestone-Ethos, Sorisole, Italy) at 200 °C. The digested sample was diluted up to 25 mL before ICP-OES analysis (iCAP 7400, Thermo Scientific).

#### Endogenous IAA Analysis

Analysis of IAA is performed using a high-performance liquid chromatography-electrospray-high-resolution accurate mass spectrometry (HPLC-ESI-HRMS) system. IAA is extracted and purified using the following protocol: the material is harvested and frozen in liquid nitrogen. It is then ground in a mortar in the presence of liquid nitrogen. To 0.1 g of the pulverized material is added 1 mL of a MeOH/H_2_O/formic acid mixture (90:9:1, v/v/v, with 2.5 mM Na-diethyldithiocarbamate) and shaken for 1 h on a Multi Reax shaker (Heidolph Instruments, Schwabach, Germany), which is then centrifuged at 13 000 rpm for 10 min in a Biofuge pico centrifuge (Thermo Fisher Scientific, Waltham, Massachusetts, USA). The supernatant is separated, and the solid is extracted again with 0.5 mL of the same extractant, 20 min of shaking, and subsequent centrifugation. The supernatants are combined, and 1 mL of the mixture is evaporated in a RapidVap evaporator (Labconco Co., Kansas City, MO, USA). The residue is redissolved in 0.25 mL of methanol/0.133% acetic acid (40:60, v/v), centrifuged for 10 min at 20 000 RCF using a Sigma 4–16K centrifuge (Sigma Laborzentrifugen gmbH, Osterode am Harz, Germany), and transferred to an injection vial.

IAA is quantified using a Dionex Ultimate 3000 UHPLC system coupled to an Exploris 120 mass spectrometry detector (Thermo Fisher Scientific, Waltham, Massachusetts, USA), equipped with an OptaMax NG ionization source, a quadrupole mass filter, a C-trap, an ion-routing multipole, and a high field orbitrap mass analyzer. A Synergi 4 μm Hydro-RP 80A 150 mm × 2 mm chromatographic column (Phenomenex, Torrance, CA) is used. The mobile phase consists of a linear gradient of methanol (A), water (B), and a 2% aqueous solution of acetic acid (C): 38% of A for 3 min, from 38% to 96% of A in 12 min, 96% of A for 2 min, from 96% to 38% of A in 1 min, followed by a stabilization time of 4 min. C remains constant at 4%. The flow rate is 0.3 mL/min, the column temperature is 35 °C, and the sample temperature is 15 °C. The injection volume is fixed at 20 μL.

The parameters of the ionization source were optimized to the following values: sheath gas flow rate, 50 au; auxiliary gas flow rate, 10 au; sweep gas flow rate, 1 au; spray voltage, 2900 V; ion transfer tube temperature, 320 °C; and vaporizer temperature, 300 °C.

The IAA standard and the deuterated internal standard 2H5-indole-3-acetic acid (D-IAA) were acquired from OlChemin Ltd. (Olomouc, Czech Republic). The detection and quantification of IAA were carried out using a product ion scan method in negative mode, using multilevel calibration curves with internal standards made from the acquired deuterated internal standards. The resolution was set to 30 000 fwhm; Q1 resolution (*m*/*z*) to 3; AGC target, standard; maximum injection time mode, auto; and RF lens at 70%. The absolute collision energy (CE) is dependent on the molecule. A mass tolerance of 5 ppm is accepted. For IAA, three fragments are analyzed. The fragment with the highest intensity is used for quantification, while the other two are used for the confirmation of the molecule. In the case of the internal standards, only the highest intensity fragment was analyzed. Instrument control and data processing were performed using the TraceFinder 5.1 EFS software. Table 1 presents the masses of IAA and internal standards as well as their main fragments and the collision energy (CE) used for fragmentation.

**Table 1:** Masses of IAA and Internal Standards

analyte	[M–H]^−^ hormone	CE (V)	[M–H]^−^ fragment 1	[M–H]^−^ fragment 2	[M–H]^−^ fragment 3
IAA	174.05605	8	130.0660	131.0697	128.0506

#### Gene Expression Analysis

To carry out the gene expression analysis, the entire root is harvested and immediately frozen with liquid nitrogen and stored at −80 °C until processing. Frozen samples, five roots per treatment, are ground with a mortar in the presence of liquid nitrogen. For RNA extraction, 80 mg of ground roots was processed with the NucleoSpin RNA Plant Kit (Macherey-Nagel, Diirefn, Germany), which includes genomic DNA digestion. RNA integrity and concentration were determined by means of an Experion Automated Electrophoresis System with RNA StdSens Chips (Bio-Rad, Hercules, CA, United States). Complementary DNA synthesis is performed using the iScript cDNA synthesis kit (Bio-Rad, Hercules, CA, United States) with 1 mg of RNA aliquots, following the kit protocol.

For real-time PCR analysis, 50 ng of cDNA and iQ SYBR Green supermix is used, which contains hot-start iTaq DNA polymerase, and the reaction is carried out in the iCycler iQ thermocycler (Bio-Rad Laboratories, Hercules, CA, United States). The following genes related to the root response to iron deficiency are studied:^28,31^*CsFRO1*, which encodes the root FCR responsive to Fe deficiency; *CsIRT1*, codifying the iron transporter IRT1; *CsHA2*, encoding a root plasma membrane H^+^-ATPase activated under Fe deficiency; and *CsFIT*, encoding the FIT transcription factor. Several genes associated with riboflavin synthesis are also measured:^28^*CsRIBA1*, *CsRIBC*, *CsPYRD*, *CsPHS1*, and *CsDMRLs*. In addition, two reference genes are used to normalize the expression of target genes: *CsTUA* (α-tubulin) and *CsCYCLO* (cyclophilin). Primer sequences and ID entries in the Cucurbits Genomics Database are gathered in Table 2.

**Table 2:** Primers and Cucurbits Genome Database ID for the Studied Genes

gene	ID	sense	primer
*CsFRO1*	Csa5G175770.1	forward	AGCGGCGGCAGTGGAATC
		reverse	GTTTGGAGGAGGTGGAGGAAGG
*CsIRT1*	Csa1G707110.1	forward	TTCGCAGCAGGTATCATTCTCG
		reverse	CACCACTCACTACAGGCAACTC
*CsHA2*	Csa1G423270.1	forward	AAGTTTCTGGGGTTCATGTGGAAT
		reverse	GTAACAGGAAGTGACTCTCCAGTC
*CsFIT1*	Csa6G148260.1	forward	TCGTTGGAGATGCAGTGTTGT
		reverse	GTCCACCTCACAATCCCTCACATTA
*CsRIBA1*	Csa4G111580.1	forward	TGAAGCCTCTGTCGACCTTG
		reverse	CGAAGCTTGGGGAGTCTAGC
*CsRIBC*	Csa6G128550.1	forward	ACTGCTTTTGACCACCAACT
		reverse	CCATTCGGCTGATTGGTTGA
*CsPYRD*	Csa6G003430.1	forward	GGCGTGCAACGACTAAGAGA
		reverse	GTGAGAAGAGGCTTCCCAGTC
*CsPHS1*	Csa1G655920.1	forward	GCCTCCTTGTTAATGCTCCA
		reverse	CGATGTCGAGATGTAACGCT
*CsDMRLs*	Csa6G366300.1	forward	GGTCCCAGGGAGCTTTGATA
		reverse	ACAACGGCATCATAGTGGGA
*CsCYCLO*	Csa7G009740.1	forward	ATTTCCTATTTGCGTGTGTTGTT
		reverse	GTAGCATAAACCATGACCCATAATA
*CsTUA*	Csa4G000580.1	forward	ACCGTTGGAAAGGAAATTGTTG
		reverse	GGAGCCGAGACCAGAACC

#### Study of the Stability Constants for Ls-Cit Heteromolecular Complex and Ls-Nit Complex

Ferric ion complexing capacities and the corresponding stability constants were determined at pH 8, following a methodology similar to that proposed by Plaza et al.^34^ Briefly, for the heteromolecular complex Ls-Cit, a stock solution of 240 mg L^–1^ Ls was prepared in 0.1 M KNO_3_, and the pH was adjusted to 8.25 mL aliquots of this stock Ls solution were mixed with 25 mL of solutions containing increasing concentrations of ferric citrate in 0.1 M KNO_3_ and pH 8, so that the final concentration of Ls in the measurements solutions was 120 mg L^–1^ and Cit-Fe concentrations varied from 0 to 1.75 mM Fe. Solutions were kept in the dark and overnight for equilibration. The following day, pH was checked in all of the solutions (confirming pH = 8) before fluorescence measurements.

For the Ls-Nit complex, a stock solution of 240 mg of L^–1^ of Ls was prepared in 0.1 M KNO_3_. Different volumes of an 18 mM Fe(NO_3_)_3_ stock solution in water were added to 25 mL aliquots of the stock Ls solution, and the solutions were placed for 2 h in an overhead shaker in the dark, being finally diluted with 25 mL of 0.2 M NaHCO_3_ at pH 8 and kept in the dark and overnight for equilibration. The following day, solutions were centrifuged (5000*g*, 5 min) to separate precipitated iron (if any), and the pH values were checked before fluorescence measurements.

Fluorescence spectra were recorded in an RF-6000 spectrofluorometer (Shimadzu, Kyoto, Japan), using an excitation wavelength (λ_exc_) of 270 nm based on a previous study of the absorption/excitation properties of Ls, showing a maximum of absorption at 270 nm. Emission was recorded between 285 and 500 nm, and the maximum of emission was located at 390 nm. Intensities of fluorescence at this maximum (λ_exc_ = 270 nm, λ_em_ = 390 nm) were used to calculate the complexing capacities and stability constants.^34^

#### Molecular Modeling of Fe Binding Sites in Fe Complexes

Molecular modeling studies were carried out using AMBER force field-molecular mechanics coupled to the ZINDO-1 semiempirical quantum method and the HF *ab initio*/6-31++G(d,p) method, implemented in Hyperchem 8.0 and Gaussian 16W, respectively.

Molecular interaction studies were carried out by calculating the stabilization energy (*E*_ST_) after geometry optimization of the system using AMBER, with atomic charges calculated using ZINDO-1. The calculation of the stabilization energy of the molecular system was performed by the subtraction of the energy of the molecular system with the molecules placed at noninteraction distances to each other (the energy of the optimized system at a noninteracting distance is equal to single point calculation; *E*_NON-INT_) and the energy of the optimized system when the molecules are placed at interacting distances from each other (*E*_INT_); *E*_ST_ = *E*_NON-INT_ – *E*_INT_. Only the final conformations corresponding to maximum values of *E*_ST_ are presented in the study.

To investigate the electronic features of Cit and Ls, we have used the HF *ab initio*/6-31++G(d,p) method. The DFT (B3LYP) method using the same basis set was also explored, but they did not reach convergence.

#### Statistical Analysis

Statistical analyses were conducted using R version 4.1.1 (R core team, 2021). Statistical differences were tested by one-way ANOVA with a significant *p* value of ≤0.05, followed by a posthoc Tukey HSD test.

## Results and Discussion

### The Ability of the Heteromolecular Complex Ls-Cit to Keep Fe in Solution in the Presence of Ca^2+^ Depends on Ls-Carbon (Ls-C)/Fe Ratio

**Figure 1:**
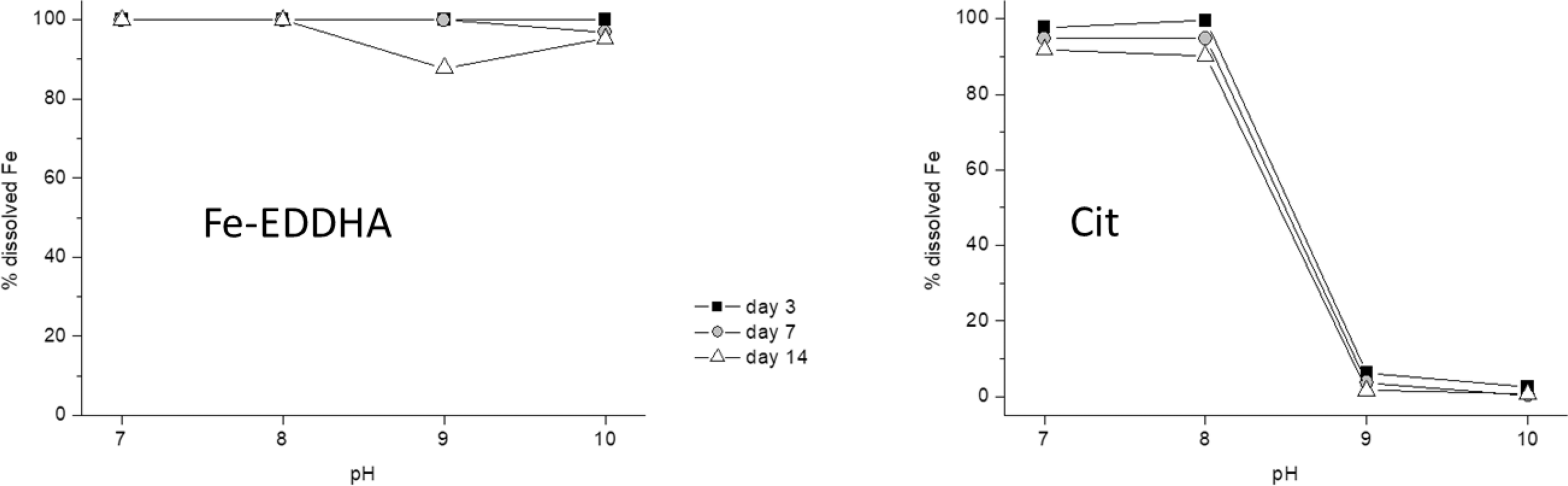
Percentage of Fe remaining in solution in the presence of 0.02 M Ca^2+^ and as a function of pH, over time, for Fe-EDDHA and Cit.

The potential efficiency of Fe-EDDHA and the Cit, Ls-Nit, and Ls-Cit complexes to maintain Fe in solution in the presence of high Ca^2+^ concentrations at alkaline pH was evaluated “in vitro” using the solubility test described in Rodriguez-Lucena et al.^33^ The results showed that Fe-EDDHA prevents Fe from precipitating (Figure 1). This result is in line with previous results and is linked to both the high stability of Fe chelation and the low affinity of EDDHA for Ca^2+^.^21^

As for Cit, the results showed that the presence of Ca^2+^ in the solution caused the precipitation of Fe at pH 9 and 10 (Figure 1). This process occurs rapidly since it is observed 3 days after the onset of the experiment. This result is in line with previous studies that showed that this chelate is not able to correct Fe deficiency and leaf chlorosis in plants cultivated in alkaline and calcareous soils when it is applied to soil, although it is efficient when applied to leaves.^1^ This fact may be explained by the displacement of iron by calcium in the complexing site due to the high Ca/Fe ratio in solution.

**Figure 2:**
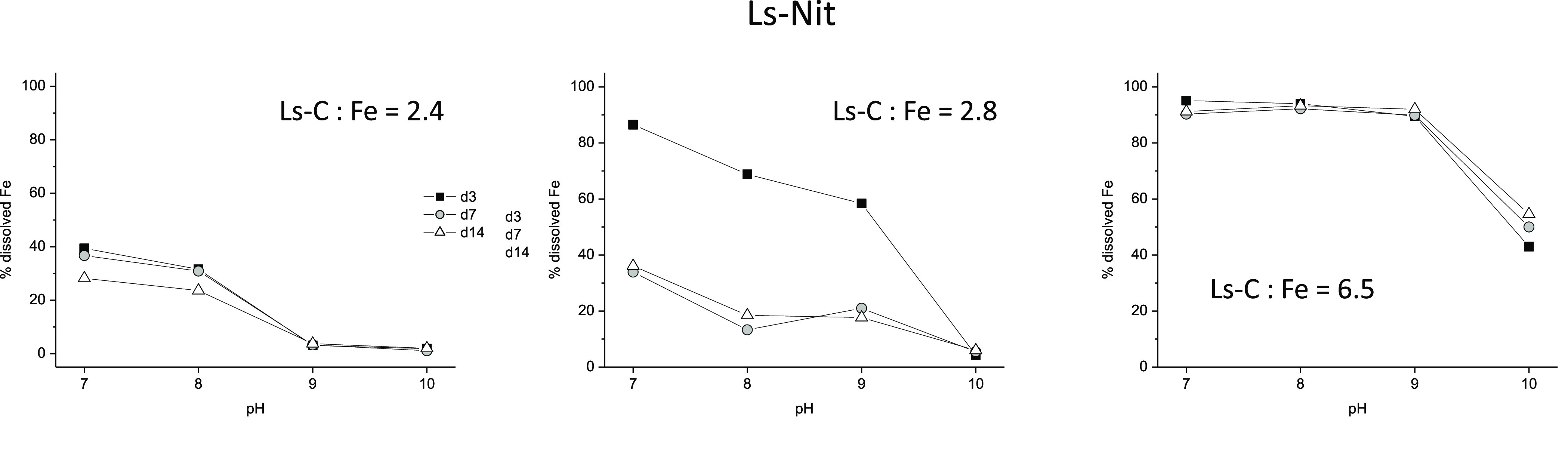
Percentage of Fe remaining in solution over time in the presence of 0.02 M Ca^2+^, as a function of pH and the Ls-C/Fe ratio, for Ls-Nit. (Ls-C is the carbon provided by Ls.)

Regarding Ls-Nit, the results showed that the Ls-C:Fe ratio influenced its stability in the presence of Ca^2+^ and depending on pH (Figures 2). Low Ls-C/Fe ratios were associated with the precipitation of Fe, mainly at high pH values (9 and 10). Higher Ls-C/Fe ratios increased the complex stability, although Fe precipitated at pH 10. There were no differences between reaction times, which indicates that Fe precipitation occurs rapidly. Previous studies have shown that Fe complexes with lignosulfonates might efficiently provide available Fe to plants growing in hydroponics but not in soil,^16,17^ mainly due to the low mobility of Ls-complexed Fe in soil.^18^ This fact seems related to Ls-Nit absorption in soil components,^18^ although complex hydrolysis cannot be ruled out.

**Figure 3:**
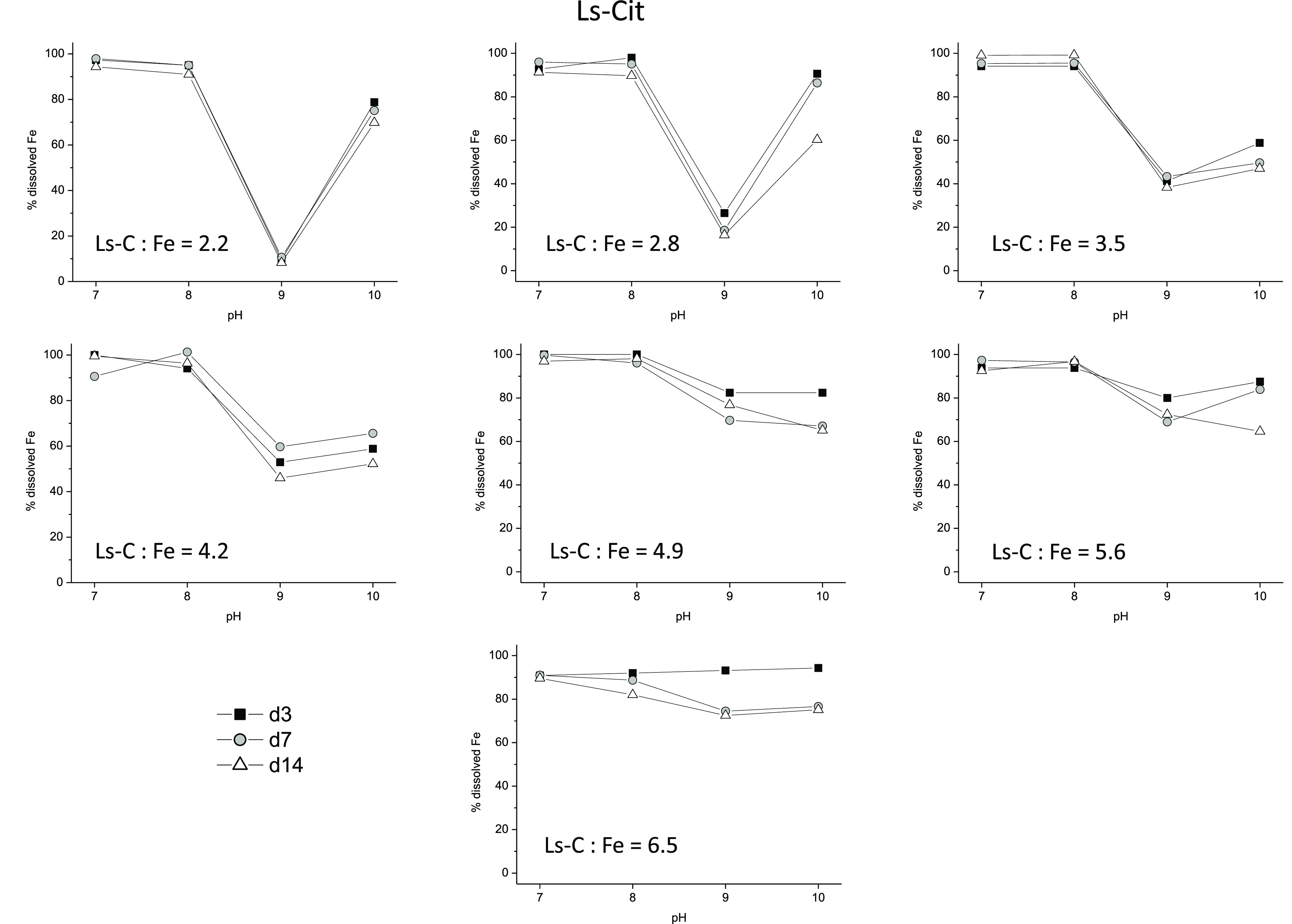
Percentage of Fe remaining in solution over time in the presence of 0.02 M Ca^2+^, as a function of pH and the Ls-C/Fe ratio, for Ls-Cit. (Ls-C is the carbon provided by LG.)

As for the Ls-Cit heteromolecular complex, the results varied with the Ls-C/Fe ratio (Figure 3). Low ratios were associated with the precipitation of Fe at pH 9 with a recovery at pH 10 (Figure 3). Medium ratios were associated with a recovery of Fe in solution at pH 9 and 10 (Figure 3), while the higher ratio showed high Fe solubility (around 80%) at pH 9 and 10. These results show that the interaction of Fe with the two complexing agents improves the stability of the complex, with respect to the Cit and Ls-Nit complexes. In principle, these results are consistent with the efficiency of this complex to correct Fe chlorosis in highly reactive soils.^21^ However, its mechanism of action might involve a direct action of the complex providing Fe to the soil solution as in the case of Fe-EDDHA and/or the involvement of Fe deficiency root responses to increasing complexed-Fe bioavailability.^21^

In this framework, studying the expression of the main root responses to increase the Fe availability in plants treated with these complexes is of great interest.

### Cucumber Plants Receiving Fe from Fe complexes (Cit, Ls-Nit, Ls-Cit) Have Root Fe Deficiency Responses Activated at the Transcriptional Level but Not at the Post-Transcriptional Level

In order to investigate the root assimilation of Fe provided by Cit, Ls-Nit, and Ls-Cit, we studied the effects of these complexes on the activation of the main Fe deficiency root responses. The treatments included Fe-EDDHA (Fe^+^) and Fe-EDDHA plus Ls (Ls) as positive controls, as well as a control that did not receive Fe (Fe^–^).

**Figure 4:**
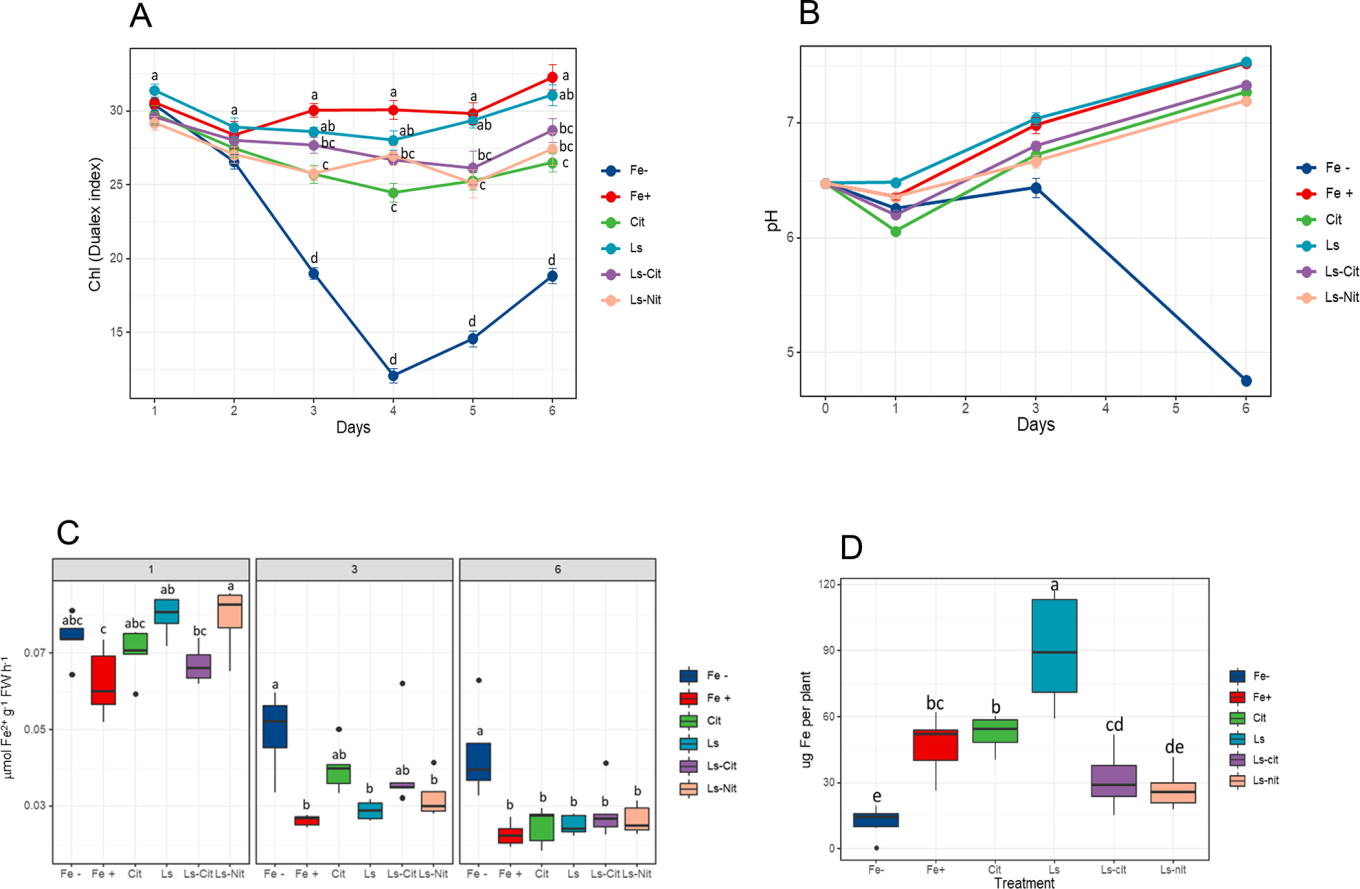
(A) Chl values of the first true leaf of the plant monitored during the 6 days of treatment. Each point represents the mean and standard error of at least five plants. (B) The pH values of the nutrient solution obtained at 1, 3, and 6 days after application of the treatments. Each point at 1, 3, and 6 days represents the mean and standard error of three, two, and one containers respectively. (C) Root FCR activity of the plant obtained at 1, 3, and 6 days after application of the treatments. Each treatment consists of five measured plants. (D) Total amount of Fe in the aerial part per plant obtained at 6 days of treatment (*n* = 5). The treatments are control without Fe (Fe^–^) or 40 μM Fe supplied as Fe-EDDHA (Fe^+^), ferric citrate (Cit), Fe-EDDHA plus Ls (Ls), lignosulfonate-iron-citrate complex (Ls-Cit), or iron-sulfonate (Ls-Nit).

The results showed that all the treatments containing Fe were associated with a concentration of chlorophyll (Chl) measured by the DUALEX index significantly higher than that of the control without Fe (Fe^–^) for the time of the study (Figure 4A). However, the positive controls (Fe^+^ and Ls) presented a higher Chl index than Cit. Ls-Cit and Ls-Nit have similar Chl values but are slightly lower than those of the positive controls, although differences were slight (Figure 4). These results indicate that plant roots can take up Fe from the different complexes and the synthetic chelate.

Under these conditions of Fe sufficiency, it is expected that the main enzymatic responses to Fe limitation, the FCR and root H^+^-ATPase activity, are not activated over the basal values for Fe treatments.^23,24^ As expected, pH values in the nutrient solution were acidified only for Fe^–^ after 3 days from the onset of treatments (Figure 4B). The Fe-containing treatments presented pH values around 7. In the case of FCR, a relative activation was observed after 1 day from the onset of treatments. This result might be caused by a latent Fe deficiency in plant seedlings that received only 1 μM of Fe during the pretreatment period. However, after 3 days, the treatments receiving Fe have lower values of FCR than Fe^–^. This fact was apparent 6 days after the onset of the treatments (Figure 4C).

Regarding the extraction of Fe by plants, the results showed that all Fe treatments extracted an amount of Fe that was higher than that of the treatment without Fe (Fe^–^; Figure 4D). Notably, the positive control containing Fe-EDDHA and lignosulfonate (Ls) extracted the highest amount of Fe. This result suggests that Ls may improve or favor Fe reduction by FCR. This fact may result from the interaction of Ls binding sites with Fe chelated by EDDHA, lowering the relative stability of Fe chelation. On the other hand, Cit presented an Fe extraction higher than Fe^+^, Ls-Cit, and Ls-Nit (Figure 4D). This fact may reflect the metabolic role of iron citrate in iron transport within the plant and its use in diverse physiological processes that involve Fe reduction in leaves.

**Figure 5:**
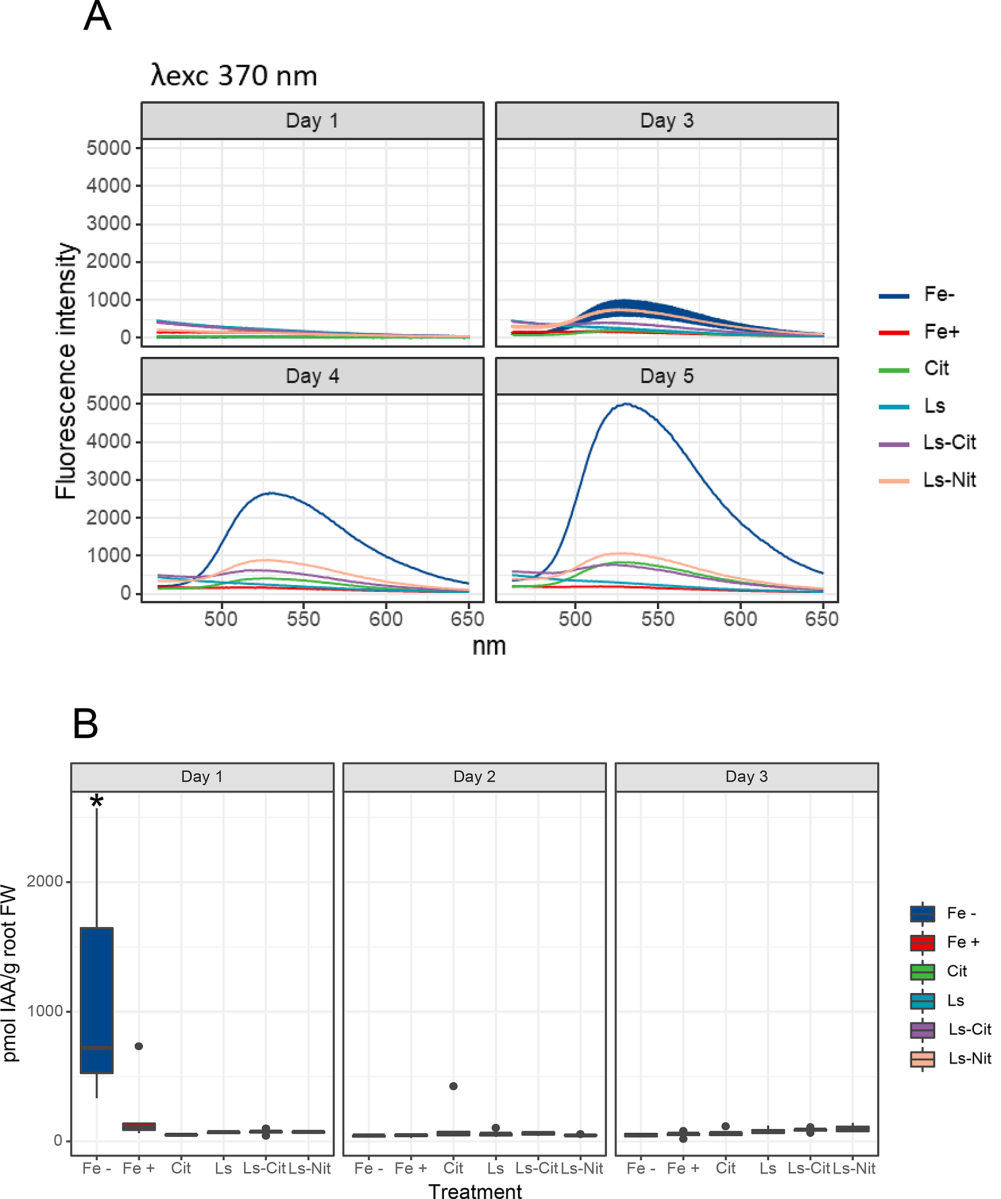
(A) Fluorescence emission spectra (excitation wavelength = 370 nm) of the nutrient solutions 1, 3, 4, and 5 days after application of the treatments; (B) IAA concentration in the root of the plants at 1, 3, and 6 days after application of the treatments (*n* = 5). The symbol * represents significant differences at *P* ≤ 0.05 (Tukey HSD post hoc test). The treatments are control without Fe (Fe^–^) or 40 μM Fe supplied as Fe-EDDHA (Fe^+^), ferric citrate (Cit), Fe-EDDHA plus Ls (Ls), lignosulfonate–iron-citrate complex (Ls-Cit), or iron-sulfonate (Ls-Nit).

Regarding riboflavin synthesis and release to the nutrient solution, the results showed an increase for Fe^–^ that was clear after 4 and 5 days from the onset of treatments (Figure 5). Positive controls did not present a release of riboflavin (Figure 5). However, Cit, Ls-Cit, and Ls-Nit presented a release of riboflavin that was much lower than that of Fe^–^ (Figure 5A).

The status of Fe sufficiency in Fe-treated plants was also reflected in the hormonal marker that we used to detect the Fe deficiency. Different studies have shown that several plant hormones control the evolvement of Fe responses in the root under Fe deficiency in a coordinated mechanism: auxin, ethylene, and NO.^23,35^ In this line, previous studies showed that Fe deficiency is associated with a prompt and transient increase of IAA in the root.^30,31^ In our study, we observe an increase in IAA in Fe^–^ after 1 day from the onset of treatments (Figure 5B). This effect seems to be triggered by the absence of Fe in nutrient solution since the Chl index did not show differences at that time (Figure 4B). However, all treatments receiving Fe showed a lower IAA concentration than Fe^–^ (Figure 5B).

All of these results, taken together, indicate that plants receiving Fe from the different synthetic chelates and complexes are growing under conditions of Fe sufficiency. Therefore, as plants receiving Fe from the different treatments did not present symptoms of Fe chlorosis in the leaves and the enzymatic root responses to Fe deficiency were not activated, the genes codifying the main actors of these responses were expected not to be activated either. However, the results showed a different scenario.

**Figure 6:**
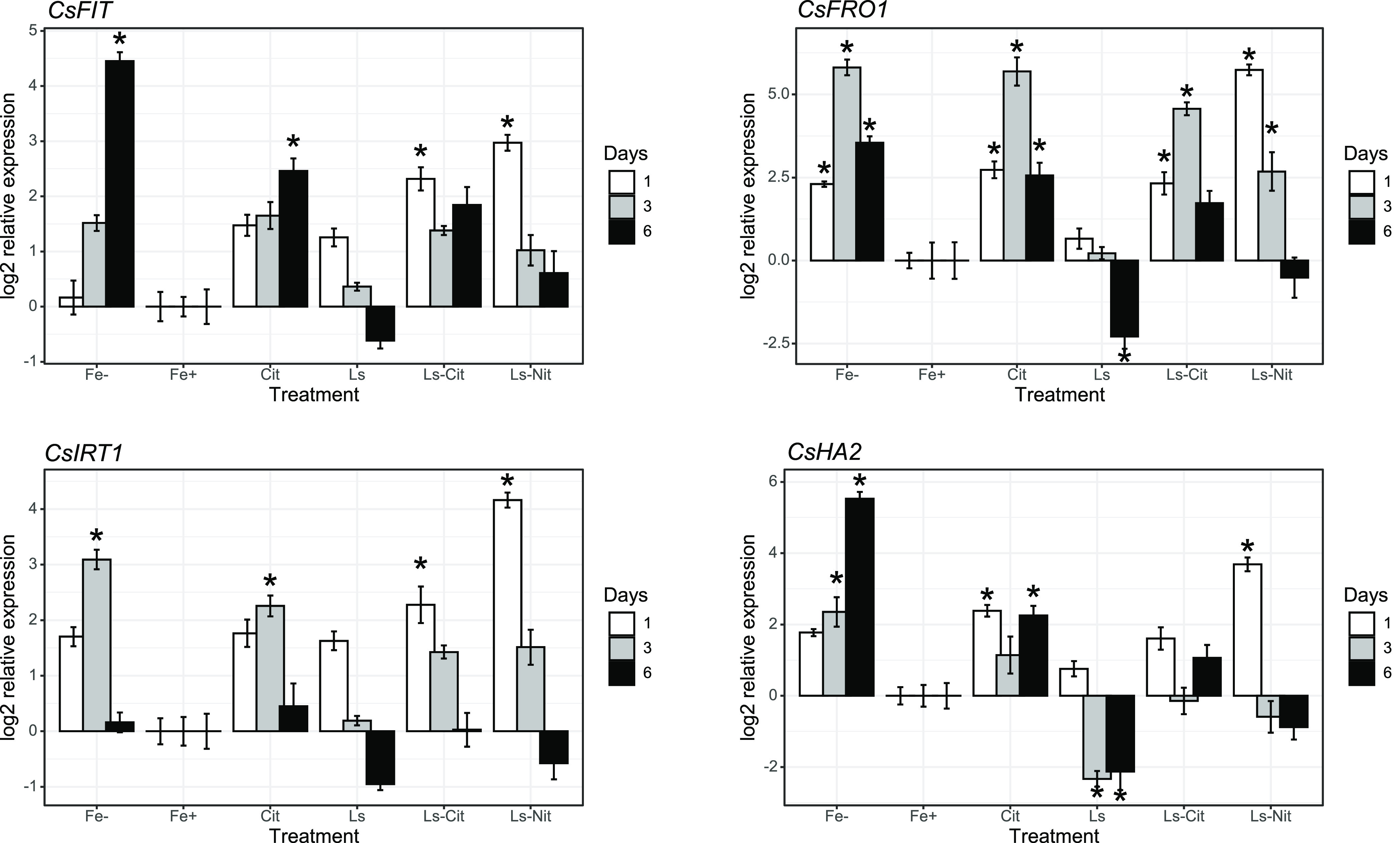
Gene expression of *CsFIT* (A), *CsFRO1* (B), *CsIRT1* (C), and *CsHA2* (D) in roots at 1, 3, and 6 days after application of the treatments (*n* = 5). The symbol * represents significant differences at *P* ≤ 0.05 compared to plants treated with Fe^+^. The treatments are control without Fe (Fe^–^) or 40 μM Fe supplied as Fe-EDDHA (Fe^+^), ferric citrate (Cit), Fe-EDDHA plus Ls (Ls), lignosulfonate–iron-citrate complex (Ls-Cit), or iron-sulfonate (Ls-Nit).

Different studies have shown that FIT is a transcription factor that regulates the expression of the main genes involved in Fe deficiency responses.^26,27^ As expected, Fe^–^ presented a significant up-regulation of this gene (*CsFIT*), principally after 6 days from the onset of treatment, when compared with Fe^+^ and Ls (Figure 6A). Unexpectedly, Cit, Ls-Cit, and Ls-Nit also showed an up-regulation of this gene despite these plants not showing Fe-deficient symptoms (Figure 6A). This scenario was also observed for *CsFRO1* and *CsIRT1*. *CsFRO1* was up-regulated in Fe^–^, Cit, Ls-Cit, and Ls-Nit treatments (Figure 6B). Also, *CsIRT1* was up-regulated for these treatments, principally for days 1 and 3, depending on the treatments (Figure 6C).

The results regarding CsHA2 were less clear than those for *CsFRO1* and *CsIRT1*. There was a clear up-regulation of this gene in Fe^–^, but the expression results for Cit and Ls-Cit were less significant (Figure 6D). Only, Ls-Nit presented a clear up-regulation after 1 day from the onset of treatments (Figure 6D).

**Figure 7:**
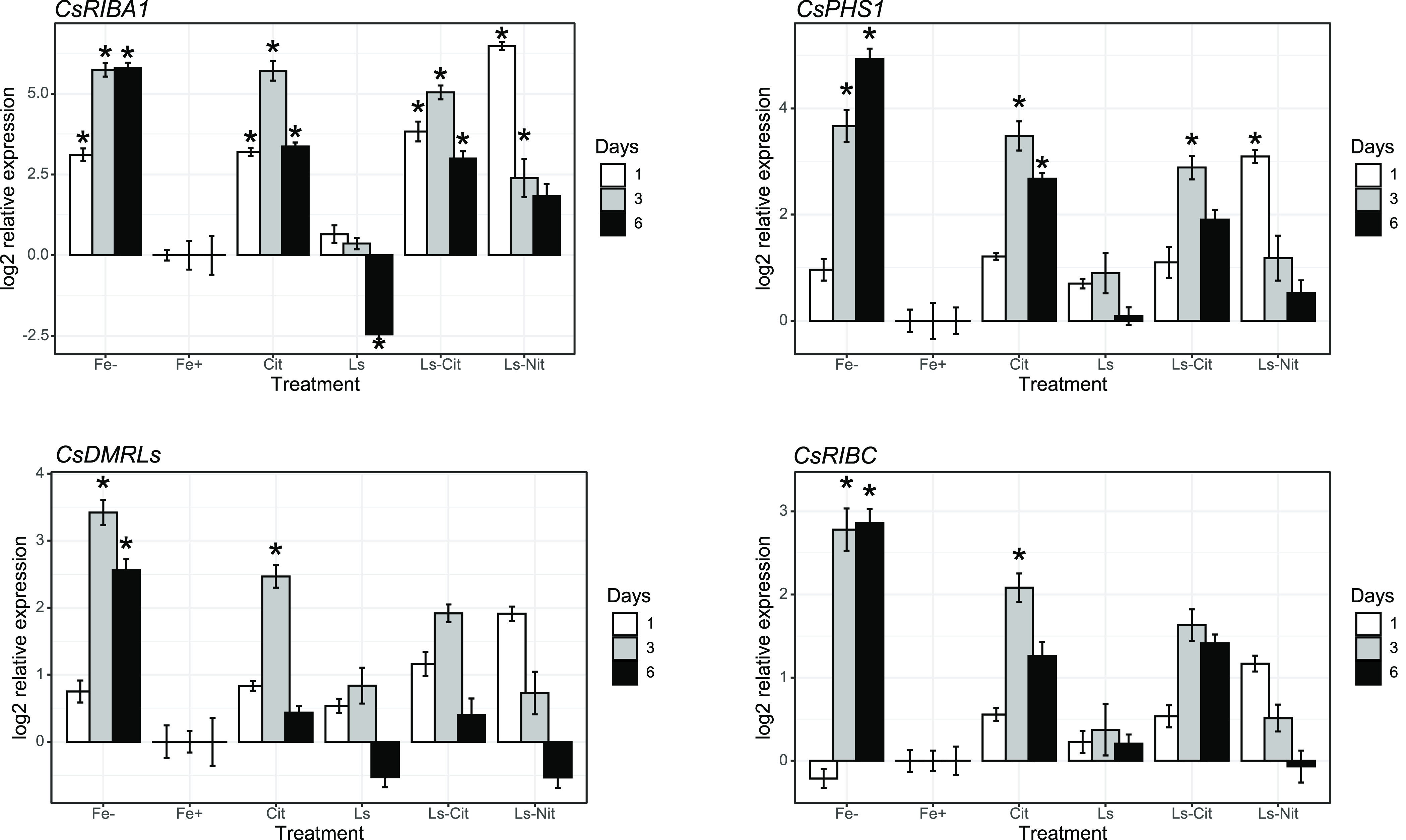
Gene expression of *CsRIBA1* (A), *CsPHS1* (B), *CsDMRLs* (C), and *CsRIBC* (D) at 1, 3, and 6 days after application of the treatments. Each treatment consists of five measured plants. The symbol * represents significant differences at *P* ≤ 0.05 compared to plants treated with Fe^+^.

Regarding the genes encoding riboflavin synthesis, only *CsRIBA1* and *CsPHS1* presented significant upregulation (Figure 7A,B). The other genes studied, *CsDRMLS* and *CsRIBC*, presented a slight up-regulation in Fe^–^ plants and plants treated with Cit (Figure 7B,C). In line with the results obtained for *CsFIT*, *CsFRO1*, and *CsIRT1*, Fe^–^ but also Cit, Ls-Cit, and Ls-Nit presented an up-regulation of these genes when compared with Fe+. It is noteworthy that in this case, a slight but measurable increase in riboflavin release to the nutrient solution was observed for the treatments with Fe complexes (Figure 5A). Other studies have observed the up-regulation of genes related to riboflavin synthesis in Fe-sufficient plants growing at alkaline pH with bicarbonate.^28^ In these studies, this fact was related to the impairment in Fe-shoot sensing under these conditions.^28^

Consequently, these results indicated that the plants receiving Fe complexed in Cit, Ls-Cit, and Ls-Nit do not have Fe deficiency responses activated at the post-transcriptional level but are activated at the transcriptional level. Many studies have shown that in many cases, the genes and the proteins involved in Fe deficiency responses do not match clearly.^36^ In *Medicago truncatula*, a good correlation of Fe deficiency responses at transcriptional and post-transcriptional levels was observed only in the case of riboflavin synthesis and release from the roots.^36^ This fact indicates that some changes observed at the transcriptional level may not be expressed at the post-transcriptional level. However, other studies showed a good correlation between gene expression, protein synthesis, and protein activity.^37^ This framework suggests that regulation at the different levels of gene expression and transduction is not the same under all experimental conditions and might involve different signaling mechanisms. In this line, our results suggest that, in the case of the different complexes employed, the regulation at the transcriptional and post-transcriptional levels involves different signaling pathways. In this sense, the main difference between Fe, which has Fe deficiency responses expressed at both levels, and the treatments with complexes is the absence of Fe outside the roots. Considering that IRT1 may also act as a sensor of available Fe outside the roots,^38,39^ the differences between Fe-deficient plants and Fe-sufficient plants receiving Fe from the complexes might be regulated by IRT1 sensing activity. Further studies are needed to explain all of these open questions. In these studies, Ls-Nit might be a good tool to develop an experimental model showing a diverse regulation of Fe root responses at the transcriptional and post-transcriptional levels.

In summary, the results show that the heteromolecular complex Ls-Cit has the main Fe-deficiency root responses activated at the transcriptional level. However, this activation is not expressed at the post-transcriptional level except for the slightly activated riboflavin secretion. This fact might be related to these complexes’ structural features and to the Fe complexation’s relative stability. In order to investigate these questions, we studied the complexation process and the structural characteristics of the binding site using fluorescence spectroscopy and molecular modeling, respectively.

### Fe Complexation in Ls-Cit Seems to Involve Different Binding Sites than in Ls-Nit

**Figure 8:**
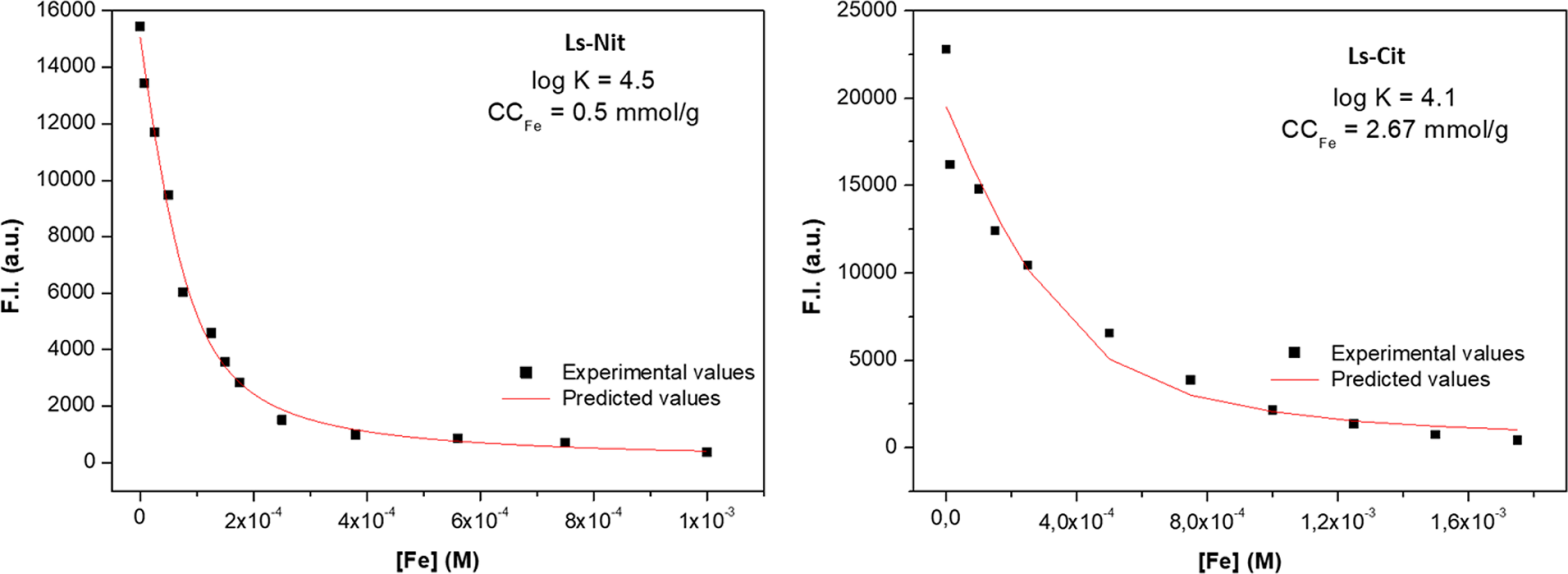
Stability constant (log *K*) and concentration of ligands involved in Fe complexation (CC_Fe_) for Ls-Nit and Ls-Cit, obtained using the fluorescence quenching approach.

The results showed that the stability of the heteromolecular complex (Ls-Cit; log *K* = 4.1) is slightly lower than that of the Ls-Nit complex (log *K* = 4.5; Figure 8). The difference between the concentrations of the ligands involved in Fe complexation for Ls-Cit (2.67 mmol g^–1^) and Ls-Nit (0.5 mmol g^–1^; Figure 8) was noteworthy. This result indicates that the features of the binding sites involved in the two complexation processes are probably different. This fact might be related to the chemical nature of the functional groups forming the binding site for each complex. In this framework, the molecular modeling of each binding interaction might help us better understand the whole process. Fluorescence emission spectra are shown in Figure S5.

### Molecular Modeling Reveals That the Main Binding Sites for Fe Complexation in Ls-Cit and Ls-Nit Are Different from Each Other and Probably Have a Diverse Affinity for Ca^2+^

**Figure 9:**
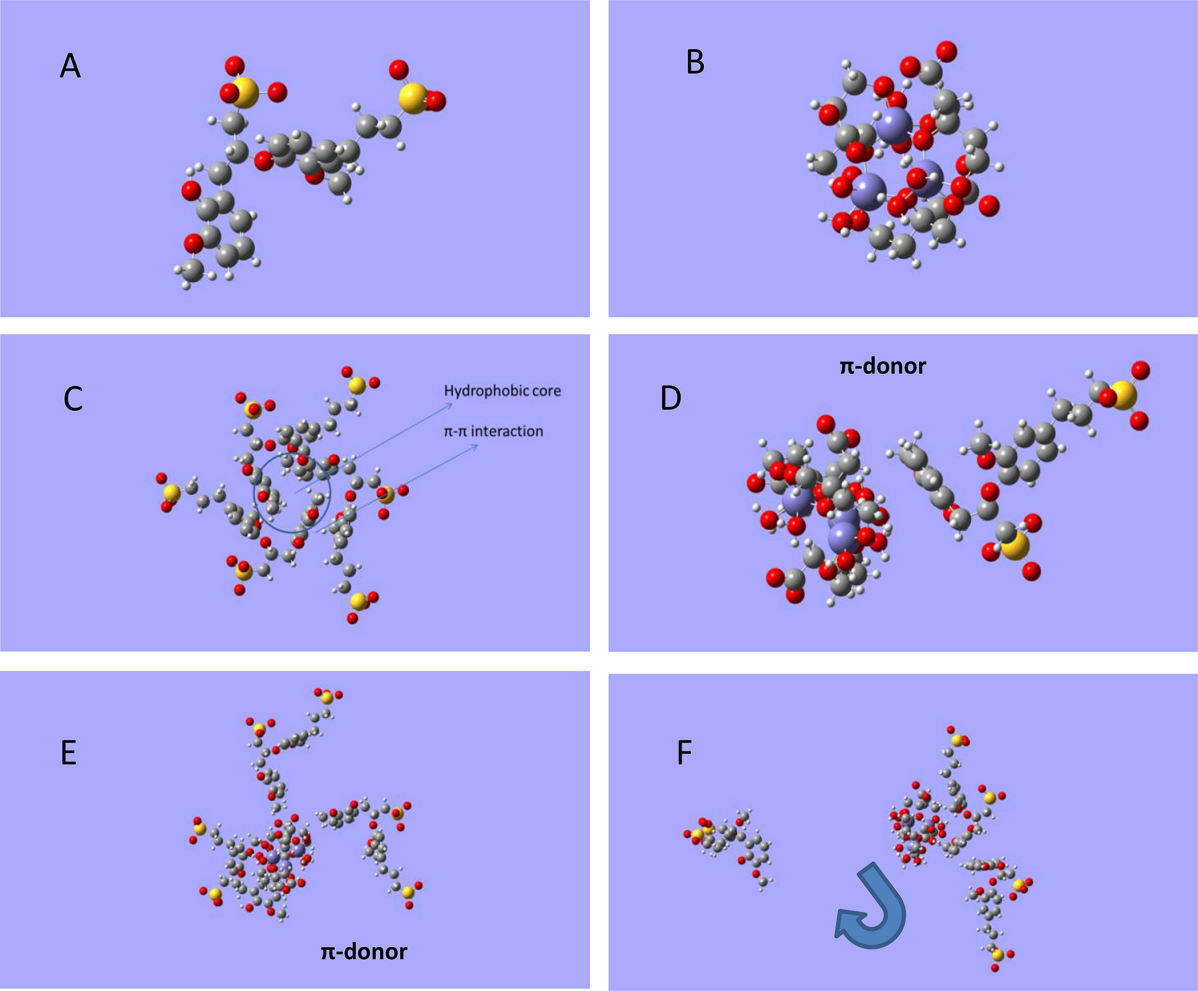
Optimized structural features of (A) LG monomer LG^2–^; (B) Cit_3_Fe_3_^3–^ chelate; (C) LG molecular aggregate LG_3_^6–^; (D) the interaction LG^2–^–Cit_3_Fe_3_^3–^; (E) the interaction LG_3_^6–^–Cit_3_Fe_3_^3–^; and (F) LG_3_^7–^–Cit_3_Fe_3_^3–^, where LG_3_^7–^ includes the ionization of a phenol group. The molecules and molecular systems were optimized with AMBER with atomic charges calculated with ZINDO-1. Color code: H, white; C, gray; O, red; Fe, violet; S, yellow.

In order to model Ls, we have employed the structure proposed for an Ls monomer (Ls^2–^; Figure 9A).^40^ This monomer was used as a basis for theoretical studies, and the Ls^2–^ structure was optimized through the following steps: structure creation and first optimization with AMBER MM/charges calculation with ZINDO-1/new geometry optimization with AMBER MM (Figure 8A). This structure was used for interaction studies. Likewise, this structure was used as the basis for the study of electronic properties employing the wave function calculated by the HF *ab initio* method in combination with the 6-31++G(d,p) basis set.

Cit was modeled using the (Cit_3_-Fe_3_)^3–^ proposed in refs (41) and (42) (Figure 9B). The sequence of calculations to obtain the optimized structure was similar to that used for Ls^2–^. The electronic features was also carried out using the HF/6-31++G(d,p) level of theory.

In the case of Ls^2–^, the molecular electrostatic potential (MEP) isosurface shows that there are two sites of interaction with negative potentials around the sulfonic groups. However, the aromatic part of the molecule has a negative-neutral electrostatic potential (Figure S1). In the case of (Cit_3_-Fe_3_)^3–^, a negative electrostatic potential surrounds the main parts of the molecule (Figure S2). These results indicate that the direct interaction of (Cit_3_-Fe_3_)^3–^ with the sulfonic groups in Ls^2–^ is not favored due to possible charge repulsion.

Several studies reported that lignosulfonate structural features involved the formation of self-assembled molecular aggregates showing a hydrophilic part in the outer region of the molecule linked to the ionized sulfonic groups and a hydrophobic aromatic core in the inner region of the supermacromolecule.^43^ The optimization of a molecular system consisting of three Ls^2–^ units produces a molecular aggregate with a distribution of ionized sulfonic groups in the outer layer of the molecule and an aromatic core in the inner part of the molecule probably stabilized by π–π ring interactions (Figure 9C). Although the AMBER force field did not include terms to parametrize π–π interactions (this process must be characterized using quantum mechanics), it works extremely well to describe this type of chemical bond.^43,44^ This fact is probably due to the relevant role of van der Waals forces in the configuration of the molecular conformation linked to these types of interaction.^44,45^ We have confirmed the interaction between Ls^2–^ molecules by calculating the system’s electronic molecular density distribution using ZINDO-1. The results showed that the electron density is distributed in the whole molecular aggregate, including the ensemble of the three Ls^2–^(Figure S3).

For a first approach to the interaction between Ls and Cit, we considered an interaction system including Ls^2–^ and (Cit_3_-Fe_3_)^3–^ structures. The energy optimization of the system rendered a complex formed by the aromatic region of Ls^2–^ and the Fe region of (Cit_3_-Fe_3_)^3–^ (Figure 9D). This interaction seems to involve a π-donor interaction from the aromatic ring to the unoccupied molecular orbitals of Fe. This type of chemical bond must be characterized by using quantum mechanics. However, AMBER described well this type of interaction.^44,45^ This interaction was confirmed by calculating the electronic density distribution of the system using ZINDO-1, which showed the interaction between the two molecular units in the molecular system (Figure S4). An interaction between the sulfonic groups and Fe in (Cit_3_-Fe_3_)^3–^ was not favored, probably due to the charge repulsion forces.

The interaction of (Cit_3_-Fe_3_)^3–^ with Ls_3_^6–^ rendered the formation of a supramolecular complex between (Cit_3_-Fe_3_)^3–^ and Ls_3_^6–^, where (Cit_3_-Fe_3_)^3–^ interacts with the three Ls^2–^ through the aromatic rings of the aromatic core (Figure 9E).

It was noteworthy that the ionization of a phenol group in Ls_3_^7–^ caused destabilization of the aggregate, affecting the (Cit_3_-Fe_3_)^3^-Ls^2–^ interaction (Figure 8F). This process might explain why the variation of pH from 8 to 9 (phenol ionization region) led to Fe precipitation in the presence of Ca^2+^ (complex destabilization due to phenol ionization), followed by a recovery of Fe complexation at pH 10 (complex reorganization; Figure 3).

**Figure 10:**
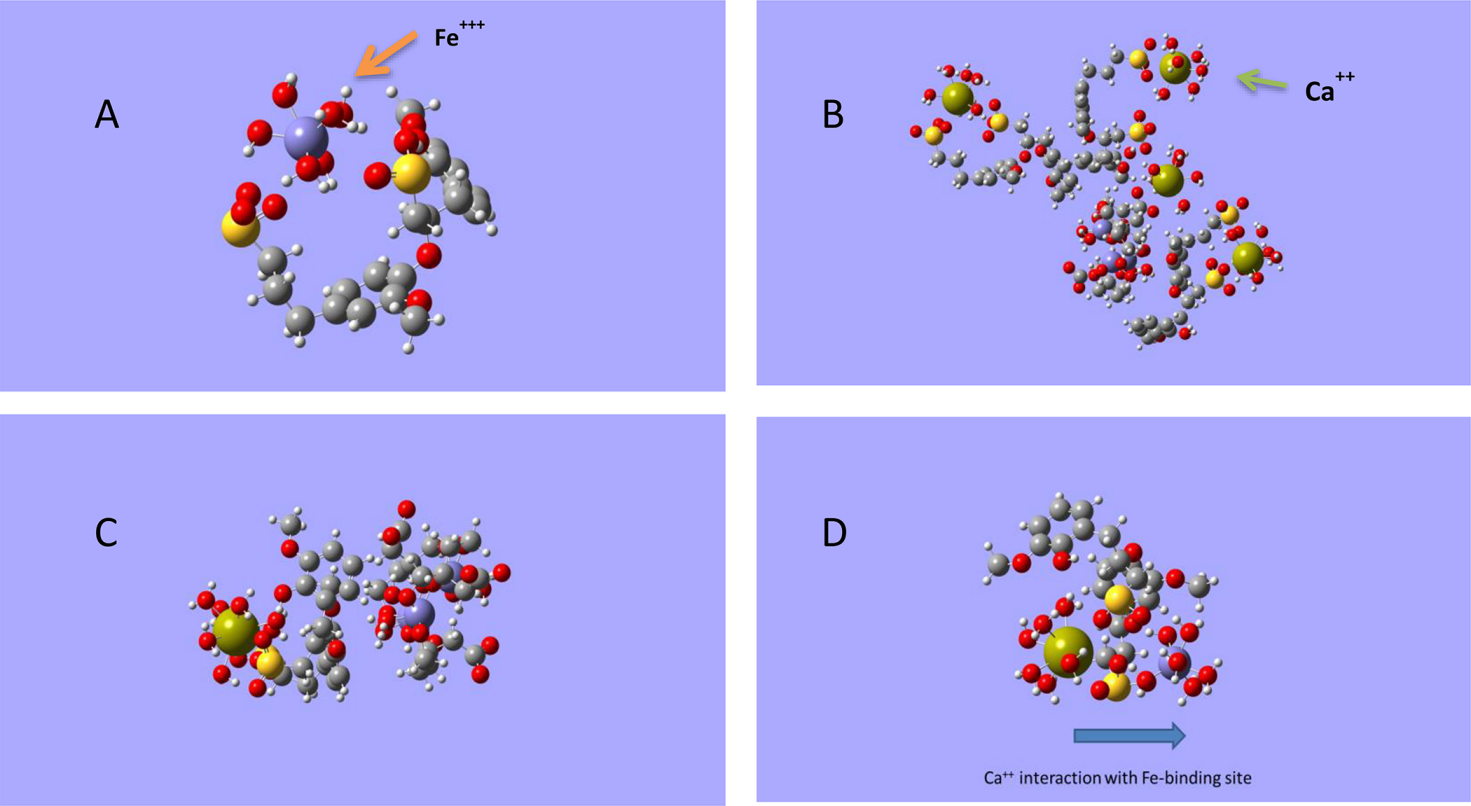
Optimized structural features of the interaction (A) Ls^2–^–Fe(H_2_O)_6_^3+^; (B) Ls_3_^6–^(Cit_3_Fe_3_)^3–^–4Ca(H_2_O)_6_^2+^; (C) Ls^2–^(Cit_3_Fe_3_)^3–^–Ca(H_2_O)_6_^2+^; and (D) Ls^2–^–Fe(H_2_O)_6_^3+^–Ca(H_2_O)_6_^2+^. The molecules and molecular systems were optimized with AMBER with atomic charges calculated with ZINDO-1. Color code: H, white; C, gray; O, red; Fe, violet; S, yellow; Ca, olive green.

In the case of the interaction of Ls^2–^ with Fe(H_2_O)_6_^3+^, Fe is directly complexed by the two ionized sulfonic groups present in Ls^2–^ (Figure 10A).

In this way, the interaction pathways leading to Fe complexation in Ls-Cit and Ls-Nit are clearly different from each other. In the case of the heteromolecular complex, a supramolecular arrangement is formed through the interaction of the aromatic rings in LG with Cit. However, the complexation of Fe by LG involves a polydentate complex with the ionized sulfonic groups.

In principle, these different structural and electronic features of Ls-Cit and Ls-Nit may affect the interaction dynamics with Ca^2+^. In this sense, it was very interesting to study the interaction of the (Cit_3_-Fe_3_)^3^-Ls_3_^6–^ and Ls^2–^-Fe(H_2_O)_6_^3+^ with Ca(H_2_O)^6+^.

In the case of the heteromolecular complex, Ca^2+^ is complexed by the sulfonic groups in Ls and the carboxylic groups in Cit_3_-Fe_3_ without directly affecting the Fe-aromatic ring interaction (Figure 10B,C). However, in the case of Ls-Nit, there is a competition between Ca^2+^ and Fe^3+^ for the sulfonic binding sites (Figure 10D). These results suggest that the heteromolecular complex will be more stable in the presence of Ca^2+^ than the Ls-Nit complex, as observed in the study of Fe in solution in the presence of Ca^2+^ and as a function of pH (Figures 2 and 3). These results suggest that the acidic free binding sites in Ls (sulfonic groups) and Cit (carboxylic groups) in the heteromolecular complex form a protective shield against the interaction with other cations, such as Ca^2+^.

Finally, the supramolecular structure of Fe complexes may affect Fe accessibility for the active center in FCR. Indeed, this fact might explain why Fe deficiency root responses are partially activated when these products are used as a substrate. In addition to this fact, the probable interaction of the supramolecule with solid components of the rhizospheric soil may have relevance. This fact could involve the participation of root exudates to mediate Fe root uptake.^25^

In summary, the results show that the lignosulfonate-based heteromolecular Fe complex has a higher capacity to maintain Fe in solution in the presence of Ca^2+^ and at basic pH than both Cit and Ls-Nit. The level of Fe solubility with the heteromolecular complex is close to that of Fe-EDDHA. However, the mechanism of Fe root uptake from the heteromolecular complex differs from Fe-EDDHA’s. In this sense, whereas Fe-EDDHA does not activate the root responses to Fe deficiency, the heteromolecular complex showed that the main Fe deficiency root responses activated at the transcriptional level but not at the post-transcriptional level. Only the root release of riboflavins was slightly promoted in the case of the heteromolecular complex. This scenario might be related to the sensing of Fe in the solution, which in turn may be associated with some features of the Fe binding sites in Fe-EDDHA and the heteromolecular complex. The molecular modeling study of the Fe binding process in the heteromolecular complex indicated that Cit (Cit_3_Fe_3_)^3–^ interacts with the hydrophobic core of the lignosulfonate and not with the sulfonic groups. This fact led to the formation of stable supramolecules with Fe atoms allocated to the inner part of the supramolecule. This fact might affect the sensing of Fe in solution and the interaction of the complexed Fe with the active center in the FCR enzyme. This configuration of the heteromolecular complex and its consequences on Fe root uptake mechanisms might explain why its action in correcting Fe deficiency involves a plant response different from that for Fe-EDDHA.

## Supporting Information Available

Molecular electronic density distribution isosurfaces and molecular electronic potential isosurfaces (PDF)

## Abbreviations

Ls-Cit: iron citrate lignosulfonate complex
Cit: iron citrate
Ls-Nit: iron lignosulfonate complex
Fe-EDDHA: ethylene diamine-N,N bis(2hydroxyphenylacetic acid) ferric chelate
Fe-HBED: hydroxybenzyl ethylenediamine ferric chelate
Fe-EDTA: ethylenediaminetetraacetic ferric chelate
HS: humic substances
Ls: lignosulfonate
Ls-C: carbon from the lignosulfonate
FCR: Fe(III) chelate reductase
Chl: chlorophyll
IAA: indole-acetic acid
HPLC-ESI-HRMS: high-performance liquid chromatography-electrospray–high-resolution accurate mass spectrometry
