# Supplementary material for: Supramolecular Arrangement of Lignosulfonate-Based Iron Heteromolecular Complexes and Consequences of Their Interaction with Ca2+ at Alkaline pH and Fe Plant Root Uptake Mechanisms
Source: J Agric Food Chem. 2023 Jul 18;71(30):11404–17. doi: 10.1021/acs.jafc.3c03474 (PMC10401718; doi:10.1021/acs.jafc.3c03474)
Supplement: Supplementary file 1 — jf3c03474_si_001.pdf [file jf3c03474_si_001.pdf]

Supporting Information

Supramolecular arrangement of lignosulfonate-based iron heteromolecular complexes and consequences on their interaction with Ca^{++} at alkaline pH and Fe plant root uptake mechanisms.

Marta Fuentes[1,2,&], German Bosch[1,2,&], David de Hita[1,2], Maite Olaetxea[1,2,], Javier Erro[1,2], Angel Mª Zamarreño[1,2], Jose Mª Garcia-Mina[1,2,*].

[1]Universidad de Navarra, Instituto de Biodiversidad y Medioambiente BIOMA, Irunlarrea 1, 31008, Pamplona, España.

[2]Universidad de Navarra, Facultad de Ciencias, Departamento de Biología Ambiental ,
Irunlarrea 1, 31008, Pamplona, España

[&] These authors contributed equally to the work.

* Corresponding author: Jose Mª Garcia-Mina, e-mail: jgmina@unav.es.

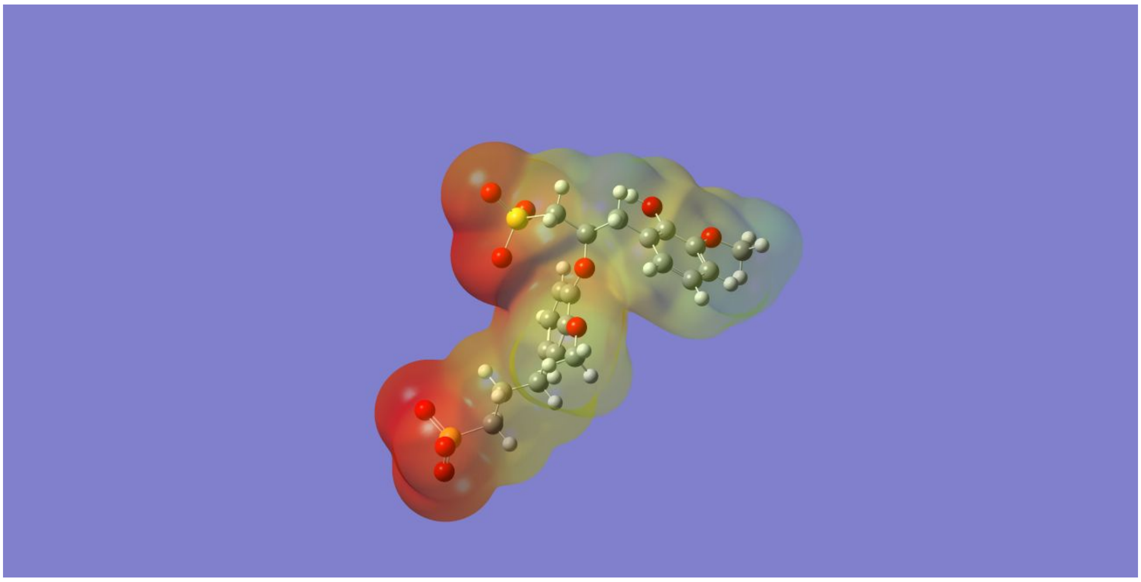

Figure S1. Molecular electrostatic potential (MEP) isosurface for the lignosulfonate monomer Ls^{2-}.

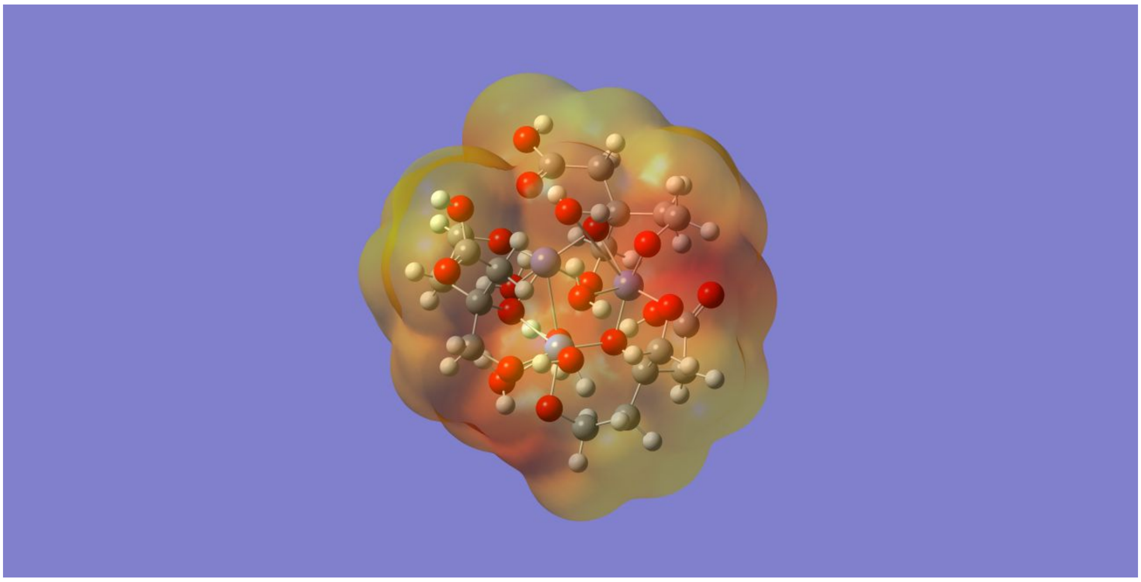

Figure S2. Molecular electrostatic potential (MEP) isosurface for the $(Cit_3Fe_3)^{3-}$ complex.

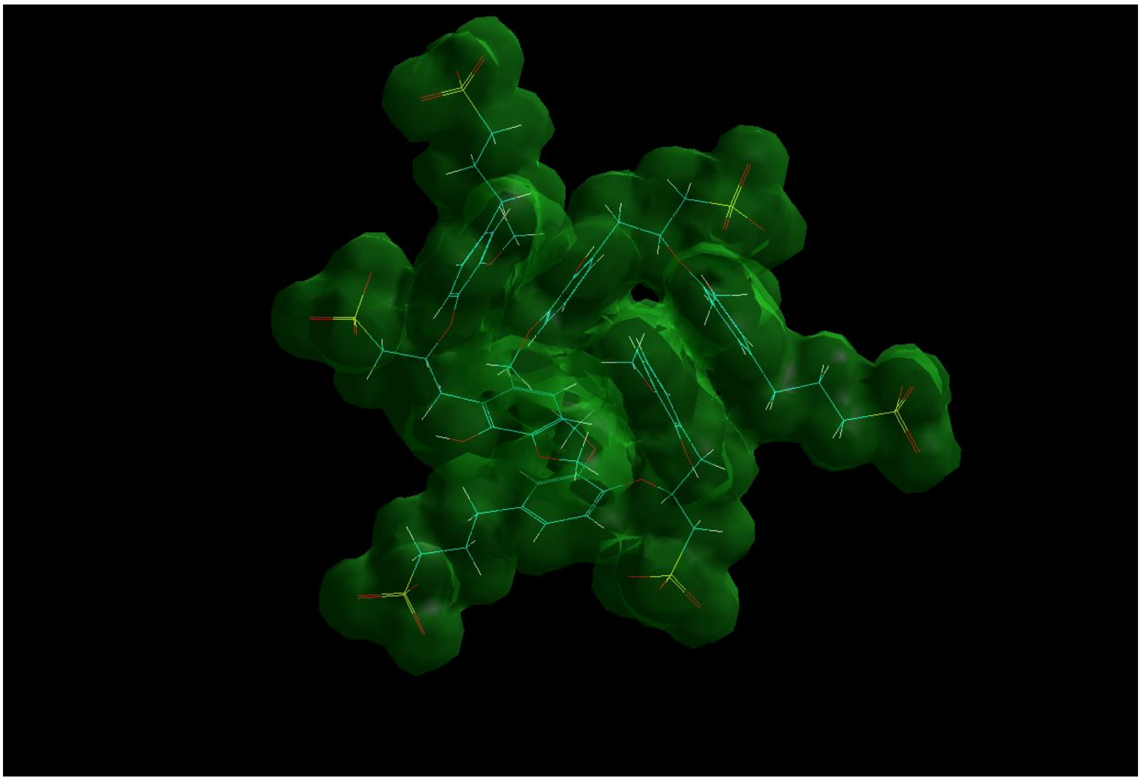

Figure S3. Molecular electronic density (MED) isosurface for the Ls_3^{6-} molecular aggregate.

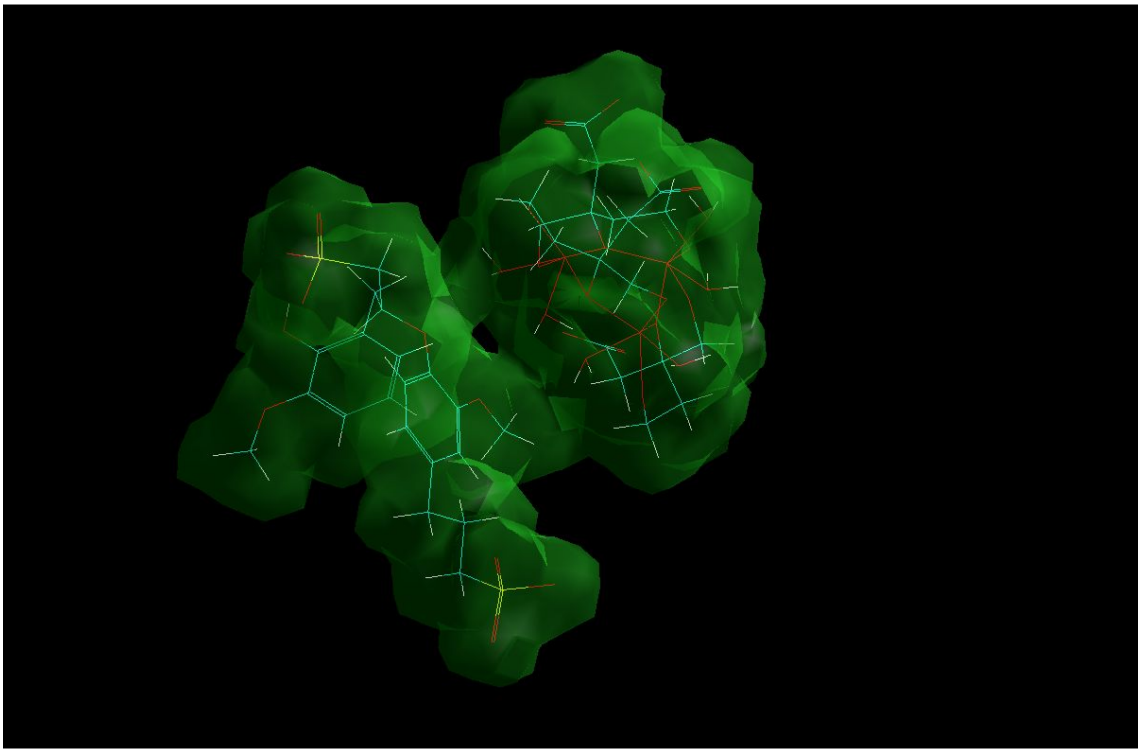

Figure S4. Molecular electronic density (MED) isosurface for Ls^{2-}-$(Cit_3Fe_3)^{3-}$ interaction.

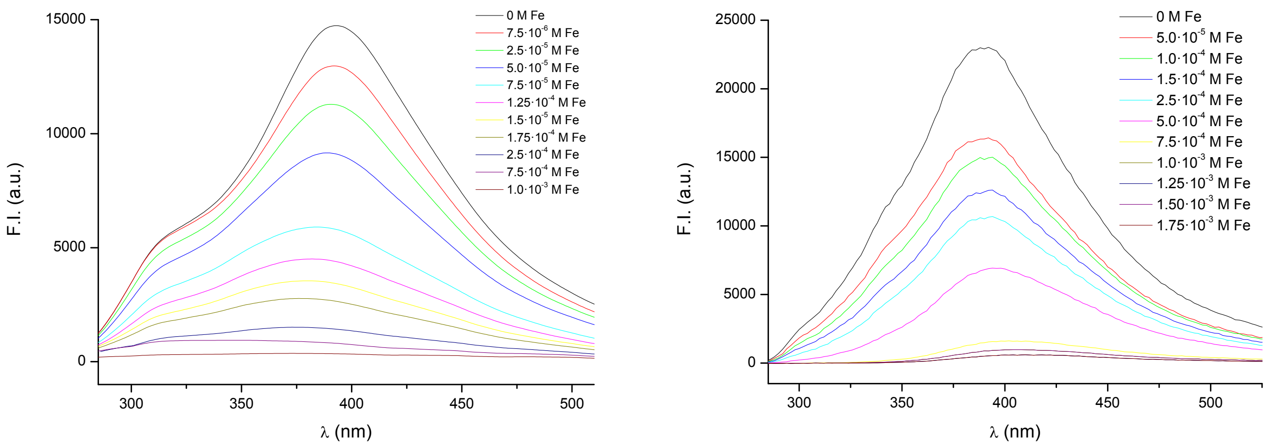

Figure S5. Fluorescence emission spectra (λ_{em} = 270 nm) of solutions containing 120 mg L^{-1} lignosulfonate and different concentrations of iron added as ferric nitrate (left) or ferric citrate (right).
